# Proposal of a new analytical procedure for the measurement of water absorption by stone. Preliminary study for an alternative to the Italian technical normative NORMAL 07-81

**DOI:** 10.1186/1752-153X-6-62

**Published:** 2012-06-27

**Authors:** Susanne Heidi Plattner, Rita Reale, Giovanni Visco, Maria Grazia Papa, Maria Pia Sammartino

**Affiliations:** 1 Department of Chemistry, University “Sapienza”, Piazzale Aldo Moro 5, 00185, Rome, Italy; 2 Free researcher,, Rome, Italy; 3 Free researcher,, Taormina, Italy

**Keywords:** Cultural heritage, Stone, Water absorption, Normative

## Abstract

**Background:**

Italian technical normative in the field of cultural heritage is often considered insufficient or not suitable in practise, therefore efforts are necessary to design new and/or improve already existing ones.

**Results:**

In this paper an alternative analytical procedure for the determination of water absorption (by full immersion) by stone material, described in the NORMAL 07-81 document, is proposed. Improvements concern methods accuracy and reduction of sample size; further also density data is obtained.

**Conclusions:**

The new procedure was applied on three different marble samples and outcomes are encouraging, but further testing is running to better understand to what extent sample size can be reduced without worsening accuracy of results, taking into account that stone is a very heterogeneous material.

## Background

Nowadays Standardisation is of fundamental importance in almost every field of human activity and technical normative is part of this quality approach. Within this framework the Cultural Heritage (CH) sector is on an outliner position and although since 2001 efforts are undertaken to produce CEN standards and although the need for standardisation is postulated periodically in International expert meetings [1], up-to-date only nine CEN standard documents have reached the publishing level [2].

Theoretically, Italy should be among the most advanced European countries, as a standardisation commission for the CH sector was set up already in 1978 (Commission NORMAL, stone materials) and in 1997 an agreement with the Italian National body for standardisation (UNI) converged the work of the NORMAL Commission into UNI. Further, in 2001 UNI presented a request to CEN for Standardisation on Conservation of Cultural Property.

However, as stated above, few CEN standards designed for CH applications have already reached the publishing level and in the meantime one must consider “adoptable” standards, for instance, for porosity measurements and water absorption on stone material [3-6]. Scaling thus down on the National level and referring to Italy, although several documents exist for stone material, for most Cultural Heritage materials technical normative lacks completely. If one tries to get an overview on Italian technical normative in the CH sector for stone material, one must consider two types of documents, those produced by the NORMAL Commission and those converged into UNI. The NORMAL standards are obviously older and the described procedures cannot be up-to-date. In fact critics were raised due to the scarce suitability of the Normal 11-85 document [7] and a proposal for improvement was made [8]. Obviously in everyday life methods different to the official ones find application and also several research trends exist. “Several pipe methods are available for measuring the water absorption: the German Karsten pipe, the RILEM pipe, the Italian pipetto and the Polish Mirowski pipe. All are NDT (non destructive testing) methods that can be used *in-situ* to evaluate the water absorption of a porous material. The water absorption of the material corresponds to its pore structure (pores accessible by water) and thus gives information about the condition of the stone. The pipe methods are used to evaluate the result of conservation; often a hydrophobic treatment” [9]. In particular Wendler and Snethlage have evaluated positively this method often used in stone conservation [10] for being cheap and relatively easy to handle, the only disadvantage seems to be the risk of the pipes falling off during the testing. As current research trends focus obviously on non-invasive measurement procedures even Cnudde et al. [11] propose a non-invasive procedure by NMR measurements for water absorption by capillarity in stone. With reference to UNI-NORMAL 11087 [12], a proposal for a non-invasive measurement procedure was made for the determination of the salt content in walls [13].

Coming back to the official Italian methods for determination of water and water vapour absorption by stone, various standards must be cited: four UNI [14-17] and three NORMAL [7,18,19] ones. Going into detail it can be noticed that the measurement of water absorption by either full immersion [18] or by capillarity [7] is carried out on relatively huge samples (for cubic shape at least 3x3x3 cm). This perhaps is not a problem if all the measurements are carried out on test samples, but if the test is conducted on original ancient material, sample’s dimensions should be kept as small as possible. Further, the experimental procedure requires continuous handling of samples thus leading to conjectural poor accuracy of the method.

In this paper an alternative procedure to NORMAL 07-81 is proposed [18], using slightly smaller sample dimensions (2x2x2 cm), and there are presented first experimental data on three different marble stones, characterised by X-ray powder diffraction (XRD). Positive outcomes are improvement of accuracy due to avoiding any sample handling during the whole immersion time as well as to the increased resolution [20] of the used analytical balance; in addition density data is obtained. Being this work part of a wider study a future comparison of this new procedure with the one described by NORMAL [18] using the same sample size will show if size really plays such an important role and to what extent it can be reduced. In fact it must be kept in mind that stone is a relatively heterogeneous material and the sample size should influence data spreading.

At this point we would like to highlight why so much importance should be given to the accuracy of water absorption measurements: it is well known that water is a key ingredient for a variety of stone deterioration processes [21-24], whose discussion goes well beyond the aim of this paper; all these processes are bound to porosity and this latter can roughly be evaluated by computing the coefficient of water absorption, as described in NORMAL 07-81 [18]. If indeed the measurement procedure of water absorption by full immersion can be improved, it can become the cheapest, simplest and user-friendly alternative to Hg porosimetry and BET because in the field of Cultural Heritage porosity measurements are mainly used to test the efficiency of protective coatings and similar by measuring a porosity variation; so, the apparent and real volumes, the total porosity and all the other parameters that can be obtained though more sophisticated techniques are rarely of practical interest. On the other hand, this perspective is very interesting and fits finely in our general research project that aims to propose procedures for evaluating the state of conservation of CH artefacts and related risk-thresholds by possibly non-invasive or at least the less invasive diagnostic techniques.

## Results and discussion

### Mineralogical characterization by XRD

All three marble samples, the white one (WM) and red ones (RM1 and RM2) were analysed with XRD. When marble sample powders were so analysed, the only detectable crystalline phase was calcite. After an acetic acid treatment secondary components became well evident: chlorite, muscovite and quartz were detected in all three samples; hematite in both red samples and K-feldspar only in RM2. The percentage of the secondary components was 1.51, 6.14 and 7.81 for WM, RM1 and RM2 respectively.

### Water absorption measurements

**Figure 1:**
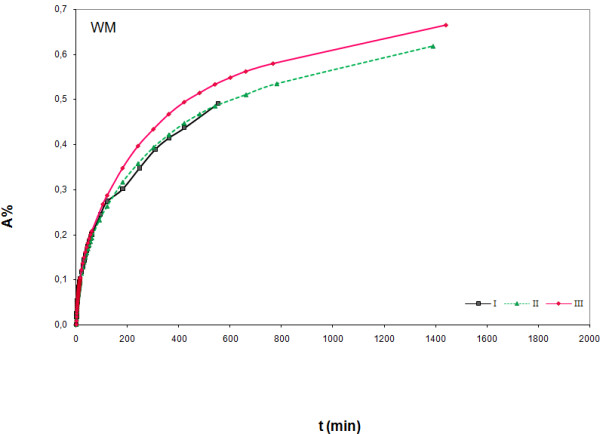
**Absorption (%) versus time (min) for white marble (WM).** Measurement results for three experiments (I-III) are shown.

**Figure 2:**
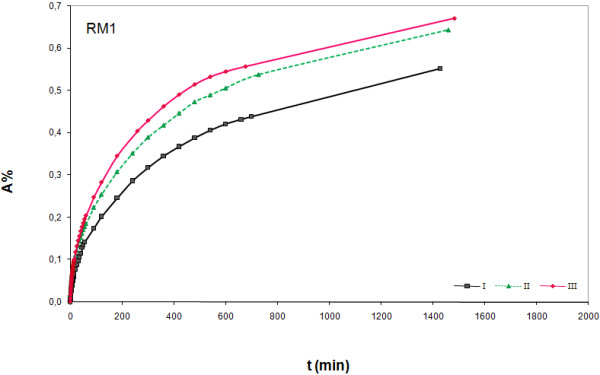
**Absorption (%) versus time (min) for red marble (RM1).** Measurement results for three experiments (I-III).

**Figure 3:**
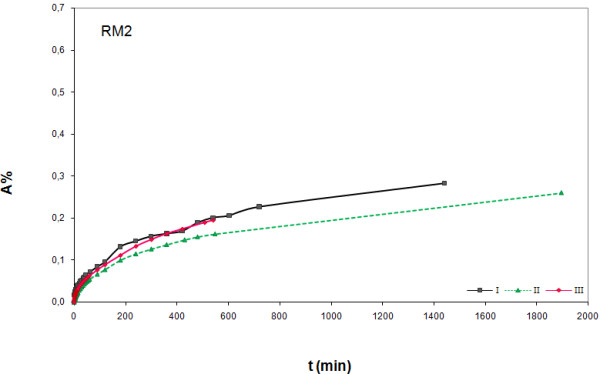
**Absorption (%) versus time (min) for dark red marble (RM2).** Measurement results for three experiments (I-III) are shown.

In Figures 1‒3 experimental results obtained with the proposed immersion procedure are shown for the three marble samples; scatter graphs reporting absorption (%) versus time of immersion were used following suggestion from the NORMAL document. As can be seen from graphs, measurements were replicated to check repeatability (trials I-III). The NORMAL document foresees test repetition using different samples of the same stone, but because of the scarce availability we repeated the tests with the same sample. Therefore we cannot draw any conclusion on the stones homogeneity, but we obtained useful data for understanding the effect of wet-dry cycles. In fact, a clear tendency of absorption increase with measurement repetition can be observed for RM1 (Figure 2) and WM (Figure 1), while for RM2 (Figure 3) absorption is lower for trial II compared to trial I, but increases for trail III. Absorption increase with measurement repetition can be due to a dissolution effect of the deionised water on the stone material, as supported by the measured weight loss (WL, see Table 1), leading to surface corrosion, but pore surface corrosion with consequent increase of inner porosity cannot be excluded. This outcome may not be considered surprising as, from a rigorous chemical point of view, deionised water can be considered a relatively strong solvent; its extremely low ionic force causes a solubility increase of any compound, being the two parameters inversely proportional.

**Table 1:** **Data on marble samples:** sample abbreviation, density, imbibition coefficient (IC %), Weight Loss (WL%) and Last Measured Weight Variation (LMWV ^0^/_00_) are given

**Sample**	**Density (gcm**^**-3**^**)**	**IC (%)**	**WL (%)**	**LMWV (**^**0**^**/**_**00**_**)**
**WM**	2.6552±0.0014	0.7125±0.0066	0.0159±0.0042	0.7432±0.1774
**RM1**	2.6525±0.0027	0.6974±0.0157	0.0149±0.0097	0.7030±0.2375
**RM2**	2.6923±0.0062	0.3069±0.0149	0.0137±0.0030	1.0102±0.0458

The different absorption behaviour of the three marble samples can be due to compositional and morphological differences. A correlation between the mineralogical composition and the water absorption capability is not very evident. Although both red samples have a very similar composition (minor components), their absorption behaviour is very different and sample RM1 behaves more like the white one (WM) rather than the other red one (RM2). Maybe K-Feldspar, detected only in RM2, is responsible for the different behaviour, but it seems more likely that morphological differences cause different absorption behaviour, as indicated by density data (see Table 1). RM1, that showed the clearest absorption increase with measurement repetition, is the marble sample with the lowest density; WM differs very slightly from RM1, while RM2 is that with the highest density. The imbibition coefficients (IC), computed as described in the NORMAL document, enhances that the absorption capability follows the order RM1 > WM > RM2 (Table 1).

As can be seen from graphs (Figures 1‒3) our measurement total time did not exceed 24 hours, but the NORMAL recommendations for measurement time were respected as for all tests the Last Measured Weight Variation (LMWV) was < 1 ^0^/_00_ (see Table 1). Further, even if not explicitly specified, a look on the table attached to NORMAL 07-81 indicates the usage of a technical balance, probably due to its higher load that allows measurement of heavy samples. In our case, thanks to the smaller stone sample size, an analytical balance was used and so measurement resolution was increased by a factor of 100; even if a 0,01 mg resolution could be used, we preferred the 0.1 one because ensuring enough suitable graph details, especially in the first part where variation is quicker.

**Figure 4:**
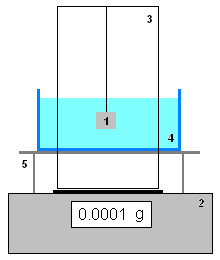
**Adsorption curve fitting for sample RM2, trial I.** The aim was the identification of the Extrapolated Final Time of Absorption (EFTA).

In Table 2 absorption data is shown after a 24 h immersion obtained by our procedure (termed A(%)_a_) and the NORMAL one [18] (termed A(%)_b_) allowing a rough comparison. The percentage difference computed in the last column is relatively high and negative for most of the measurements. This can mean that the absorbed water volume was generally underestimated when measured following the instructions of the NORMAL document [18]. Indeed measurement results must here depend on how the stone cubes are passed on the absorbing cloth (pressure, contact time) before weighing as well as on the humidity level of the latter. Obviously trained operators may produce data with better precision and accuracy than we did, but it is indisputable that by avoiding continuous sample handling the methods precision and accuracy is improved so making our procedure more robust and almost "idiot proof". On the other hand, in our procedure an “in defect error” comes from the error on the first experimental value, due to the initial movement of the sample. What was lacking in this preliminary tests in our equipment was a data acquisition software; by using such a simple tool, also clerical errors can be avoided and the final part of the curve can be increased in detail. Lacking of this equipment leads to few data points after the first 12 hours of measurements. However, very long-lasting measurements with a balance are affected also by the instruments drift, which in our case ranged from 0.3 to 0.5 mg·h^-1^ (based on a 24 h evaluation), thus decreasing the significance of measurements lasting longer than 24 hours. To overcome this problem we tried curve fitting in order to identify the Extrapolated Final Time of Absorption (EFTA); as an example in Figure 4 the curve obtained on RM2, trial I, is shown, while all results are summarised in Table 3. Unfortunately, data spreading is too wide for being a suitable method. The availability of a complete detailed curve can allow to obtain, through extrapolation, also a more reliable first value so decreasing the above said error.

**Table 2:** **Comparison of experimental data obtained with different measurement procedures**^**1**^

**Sample**	**measurement trial n°**	**A(%)**_**a**_	**Mean A(%)_a_**	**A(%)**_**b**_	**Mean A(%)**_**b**_	$\frac{A{\left(\%\right)}_{b}-A{\left(\%\right)}_{a}}{A{\left(\%\right)}_{a}}\;100$
**WM**	I	0.621		0.514		-17.2
	II	0.618	0.635±0.026	0.560	0.580±0.078	-9.4
	III	0.665		0.666		0.1
**RM1**	I	0.552		0.560		1.4
	II	0.644	0.622±0.062	0.570	0.567±0.006	-11.5
	III	0.671		0.570		-15.1
**RM2**	I	0.217		0.213		-1.8
	II	0.260	0.239±0.029	0.177	0.198±0.019	-31.8
	III	0.272		0.203		-25.5

**Table 3:** **Measured geometric volume (V**_**geom**_**), apparent volume (V**_**app**_**), pore volume percentage (V**_**pori**_**) and Extrapolated Final Time of Absorption (EFTA) computed after curve fitting for the three samples**^**2**^

**Sample**	**V**_**geom**_**(cm**^**3**^**)**	**V**_**app**_**(cm**^**3**^**)**	**V**_**pori**_**(%)**	**EFTA (min)**		
				**Trial I**	**Trial II**	**Trial III**
**WM**	7.90	7.72 ± 0.00_2_	1.60 ± 0.13	*	1854	2401
**RM1**	7.90	7.53 ± 0.00_4_	1.59 ± 0.17	2403	1673	2239
**RM2**	7.60	7.42 ± 0.02	0.72 ± 0.03	1566**	2880	2132

## Experimental

A short description of the Normal 07-81 [18] document may be useful.

Regularly shaped samples, generally cubic samples with edge length ranging from 3 to 5 cm or a surface/volume ratio ranging from 2 to 1.2 cm^-1^ must be considered and for each analysis at least 3 samples are needed. For the first test, each sample must be dried at 60 ± 5 °C, then cooled down in a disessicator and this treatment is repeated until a constant weight (M ± 0.1^0^/_00_) is reached. Then each sample is immersed in water, covered by at least 2 cm water and at established times, chosen at the best, samples are extracted from the bath, packed on a humid cloth and weighed. Measurements at 1 and 8 h immersion time and then daily are imperative and the measurement must be stopped when the difference between two successive weighings is ≤0.1^0^/_00_ M. At the end of the measurement the sample is dried again until a constant weight is reached.

The adsorbed water as percentage expression (A%) at each established times must be calculated (see formula 1) and a graph plotted as a function of immersion time. Further, the imbibition coefficient must be also calculated (see formula 2).

(1) $$A\%=\frac{w_{\mathrm{wi}}-w_{d}}{w_{d}}\cdot 100$$

where W_wi_ is the weight of the sample at immersion time i while W_d_ is the initial dry weight

(2) $$\text{IC=}\frac{w_{\text{lm}}{-\text{w}}_{\text{fd}}}{w_{\text{fd}}}\cdot 100$$

where w_lm_ is the last measured weight and w_fd_ is the weight of the sample dried after measurement ending.

### Stone samples

Water absorption measurements by full immersion were carried out on three different cubic marble samples (WM, RM1 and RM; all 2x2x2 cm), coming from an ancient CH site. For each sample measurements were repeated for a total of three trials for sample.

Cubes were measured with a caliber (0.05 mm resolution) and the obtained geometric volumes are reported in Table 3.

The samples mineralogical composition was analysed by X-ray powder diffraction (XRD), as compositional variations may explain different absorption behaviour. XRD analysis was carried out by a Seifert diffractometer MZIV using a X-ray source with a copper anode. Powdered samples were so analysed and after a chemical treatment with acetic acid 0.3 M (about 1 g for RM1 and RM2 or about 2 g for WM powder was stirred in 100 ml of the acidic solution for 15 h and then filtered) in order to remove the main constituent, calcite, whose signal hid that of the others.

### Equipment and procedure for water absorption measurements

#### Equipment

**Figure 5:**
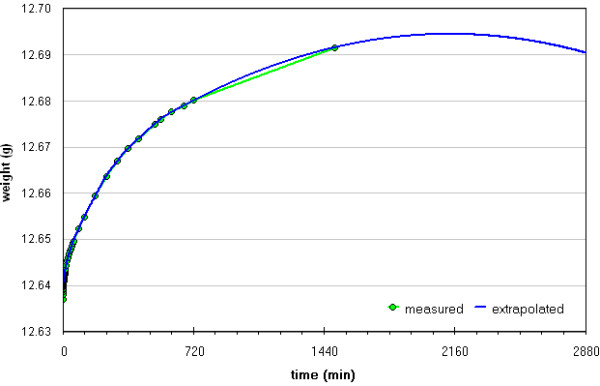
**Scheme of the measurement equipment.** An analytical balance (2) with a special weighing plate with a metallic construction (3) is used to hang the sample (1) into a liquid bearing beaker (4); the latter is placed on a small table (5) whose feet stand around the weighing plate.

Stone samples were weighed on an analytical balance (Gibertini E50S) equipped with an accessory for density measurements (see Figure 5): a special weighing plate with a soldered metallic structure from which the sample, fixed on a hook, was hung into a glass beaker filled with deionised water; the beaker was placed on a small table whose feet stood around, and not on the weighing plate.

#### Procedure

Samples were subjected to cycles of heating at 105 °C to eliminate absorbed water followed by cooling in a desiccator and weighing, until a constant weight was reached (initial dry sample weight: w_d_). The NORMAL 07-81 procedure indicates a heating temperature of 60 ± 5 °C in order to not interfere with any organic material applied on the stone in the past. As our samples were surely not subjected to such a treatment we preferred a higher heating temperature, according to general laboratory practice, in order to speed up the procedure.

The balance density equipment was mounted and zero point was set with the hook immersed in the beaker filled with deionised water; the stone sample, stored in desiccator after the previous weighing to obtain the dry mass, was then hung on the hook and completely immersed into the water bearing beaker (Figure 5). At this point the sample was not touched anymore and the water volume penetrating into it was evidenced by the increasing weight readings of the balance. Readings were registered for 24 hours at a varying frequency (every 30s for the first 15 min, then every 5 min and after the first hour at longer intervals). The difference of weight readings at immersion time “0” and immersion time “i” allows to obtain the absorbed water volume (which corresponds to the wet (w_wi_) and dry (w_d_) sample weight difference (w_wi_ - w_d_) of the above given formula 1 [18]) for the percentage absorption (A(%))

Finally graphs were drawn where the amount of absorbed water, expressed as Absorption (%), was plotted against immersion time. Blank tests were performed, by monitoring the weight variation in absence of sample, in order to evaluate the balance drift and any other effect rising from the presence of the hook and from an eventual water evaporation.

The weight loss, probably due to marble corrosion was also calculated as difference between the initial and final dry weights (Table 1).

The imbibition coefficient (IC) was calculated as well as described above in formula 2 and is reported in Table 1

Density data of stone samples (Table 1) was computed considering sample weight measured in air and in water, with the density measurement accessory and taking into account density of air and water related to their temperature [20,25].

A rough comparison of our procedure with the one described in the NORMAL document was carried out. After 24 hours of immersion the stone sample was extracted from water, passed on humid filter paper in order to absorb excess water and weighed as indicated by the normative; the above described formula 1 for percentage absorption was applied thus allowing for each marble sample a comparison of the results obtained with the two different procedures for water absorption after a 24 hour immersion.

Curve fitting for data extrapolation (EFTA) was carried out by using a bicubic SPline with XLXTR FUN software, an add-in for excel [26]. The same software was used to obtain the initial wet weight mass from which we roughly estimated the apparent volume and the pore volume. The apparent volume differs noticeably from the geometric one (see Table 3); this difference can be partially due to surface open porosity and surface roughness the volume of which is computed in the geometric volume.

## Conclusions

In this paper a new analytical procedure is proposed as concerns water absorption by full immersion of stone material. The core part of the procedure is an accessory for density measurements of an analytical balance. The procedure is easy and user-friendly and allows high accuracy due to avoiding of sample handling during measurement, as the absorbed water volume is derived directly from balance readings during sample immersion; instead the current Italian technical normative NORMAL 07-81 foresees immersion of stone samples, extraction, soft padding, weighing and re-immersion in order to follow water absorption in time. Well detailed curves and very accurate single values were obtained thank to the high resolution of the analytical balance, as well as from avoiding sample’s handling; on the other hand, basing on so few data, nothing can be said on the accuracy of A%, IC and so on.

The observed different absorption behaviour of the three marble samples is more plausibly due to morphological differences rather than compositional ones. Understanding this behaviour is surely important, but it goes beyond the aim of this paper. However, further research, requiring more samples and additional analytical techniques will be carried out for knowledge acquisition.

Further testing are indeed required also to decide on best sample number and size in order to reach a compromise with the criterion for low invasiveness. An acquisition software is currently tested to avoid clerical errors and to improve results by increasing the number of measurement points. In any case the balance drift must be considered for long lasting measurements. Our attempt to estimate the time necessary to reach maximum absorption (EFTA) by curve fitting and extrapolating data has not been so successful, as data is spread too widely for each sample; even if curve fitting was good in most cases, explaining such results as a real change of the porosity characteristics during the tests would be not significant from the statistic point of view.

## Endnotes

^a^The percentage absorption, A(%), of water by three marble samples after a 24 h immersion obtained by the here proposed new method (A(%)_a_) is compared to that obtained with the NORMAL method (A(%)_b_). The difference, calculated as percentage of A(%)_a_, is computed in the last column.

^b^As samples are subjected to dissolution phenomena while carrying out measurements, absorption curves and consequently EFTA are different for each trial for the same sample. (* fit impossible; **bad fit).

## Competing interests

The authors declare that they have no competing interests.

## Authors’ contribution

MGP cared reference researches and the English language. All the other authors equally contributed to the research. All authors read and approved the final manuscript.
